# Dose-dependent effects of selenite (Se^4+^) on arsenite (As^3+^)-induced apoptosis and differentiation in acute promyelocytic leukemia cells

**DOI:** 10.1038/cddis.2014.563

**Published:** 2015-01-15

**Authors:** S Wang, Z Geng, N Shi, X Li, Z Wang

**Affiliations:** 1 State Key Laboratory of Coordination Chemistry, School of Chemistry and Chemical Engineering, Collaborative Innovation Center of Advanced Microstructures, Nanjing University, Nanjing, China

## Abstract

To enhance the therapeutic effects and decrease the adverse effects of arsenic on the treatment of acute promyelocytic leukemia, we investigated the co-effects of selenite (Se^4+^) and arsenite (As^3+^) on the apoptosis and differentiation of NB4 cells and primary APL cells. A 1.0-*μ*M concentration of Se^4+^ prevented the cells from undergoing As^3+^-induced apoptosis by inhibiting As^3+^ uptake, eliminating As^3+^-generated reactive oxygen species, and repressing the mitochondria-mediated intrinsic apoptosis pathway. However, 4.0 *μ*M Se^4+^ exerted synergistic effects with As^3+^ on cell apoptosis by promoting As^3+^ uptake, downregulating nuclear factor-*к*B, and activating caspase-3. In addition to apoptosis, 1.0 and 3.2 *μ*M Se^4+^ showed contrasting effects on As^3+^-induced differentiation in NB4 cells and primary APL cells. The 3.2 *μ*M Se^4+^ enhanced As^3+^-induced differentiation by promoting the degradation of promyelocytic leukemia protein–retinoic acid receptor-*α* (PML–RAR*α*) oncoprotein, but 1.0 *μ*M Se^4+^ did not have this effect. Based on mechanistic studies, Se^4+^, which is similar to As^3+^, might bind directly to Zn^2+^-binding sites of the PML RING domain, thus controlling the fate of PML–RAR*α* oncoprotein.

Acute promyelocytic leukemia (APL) is a subtype of human acute myeloid leukemia.^1^ The promyelocytic leukemia protein–retinoic acid receptor-*α* (PML–RAR*α*) fusion protein, which is generated from a specific chromosome translocation t(15;17)(q22;q21), is the key driver of APL leukemogenesis.^2^ Arsenic trioxide (ATO), which has been successfully used in the treatment of APL, induces the catabolism of PML–RAR*α* oncoprotein.^3^ ATO is one of the primary therapeutic agents for APL, but organ toxicity, especially for the liver and kidney, causes excessive pain for patients.^4, 5^ Studies on the toxicity of arsenic suggest that ATO metabolism increases its toxicity because of oxidative damage and generation of more toxic metabolites, including monomethylarsonous acid and dimethylarsinous acid.^6, 7, 8, 9^ Thus, identifying new therapeutics to decrease the adverse effects of ATO is necessary.

ATO induces both apoptosis and differentiation in human APL cells.^10^ Apoptosis is an ordered cascade of enzymatic events.^11^ Studies on the mechanism of ATO-induced apoptosis in APL cells suggest that ATO promotes apoptosis through the mitochondria-mediated intrinsic pathway that is induced by oxidative stress and regulated by Bcl-2 family members.^10, 12, 13^ ATO can also induce apoptosis by inhibiting the nuclear factor-*к*B (NF-*к*B) pathway that regulates the expression of various survival proteins.^14, 15^ In addition to apoptosis, ATO can induce the differentiation of APL cells by degrading the PML–RAR*α* fusion protein and activating the retinoic acid signaling pathway.^10, 16^ Zhang *et al.*^16^ reported that ATO induced the degradations of PML and PML–RAR*α* oncoprotein by directly binding to PML. PML is a zinc-finger protein with a Cys-rich motif that contains a RING domain. The PML RING domain (PML-R) contains two Zn^2+^-binding sites (ZFs) and requires Zn^2+^ for autonomous folding.^17^ The conserved Cys12, Cys29, and Cys32 residues in PML-R-ZF1, and Cys24, Cys40, and Cys43 residues in PML-R-ZF2 are the binding sites for trivalent arsenic.^16^

Selenium is an essential nutrient element that shows chemopreventive effect and anticancer potential.^18^ Li *et al.*^19^ suggested that high dose (5.0–20 *μ*M) of selenite (Se^4+^) could induce the accumulation of reactive oxygen species (ROS) and the apoptosis of NB4 cells. Subsequently, Zuo *et al.*^20^ and Guan *et al.*^21^ confirmed that high concentrations of Se^4+^ induced the apoptosis of NB4 cells through an ROS-mediated pathway. However, the accumulation of ROS could induce adverse effects to noncancer tissues by causing oxidative damages.^22^ For cancer treatment, we attempt to increase the anticancer efficacy while decreasing the adverse effects. Thus far, few studies have investigated the effects of 2.0–4.0 *μ*M Se^4+^ on the apoptosis and differentiation of human APL cells. Selenium exerts its biological functions dose-dependently.^22^ In addition, selenium has chemical properties and metabolic fates similar to those of arsenic. In consideration of the typical characteristics of ATO in the treatment of APL, we hypothesized that 2.0–4.0 *μ*M Se^4+^ might induce some interesting changes in APL cells, such as differentiation and the degradation of PML–RAR*α*.

Combination therapy is widely used in cancer treatment. The relationship between selenium and arsenic is complex. Selenium and arsenic act as metabolic and toxic antagonists.^23^ Combining a low concentration of Se^4+^ with ATO might decrease the toxicity and increase the curative potency of ATO in the treatment of APL. Thus, it is of great significance to evaluate the effects of combining selenium with arsenic on the apoptosis and differentiation of human APL cells.

In this study, we found dose-dependent contrasting effects of Se^4+^ on arsenite (As^3+^)-induced apoptosis and differentiation in NB4 cells and primary APL cells. A 4.0-*μ*M concentration of Se^4+^ enhanced As^3+^-induced apoptosis through downregulation of NF*-к*B and activation of caspase-3, but 1.0 *μ*M Se^4+^ failed to elicit these effects. At 2.0–4.0 *μ*M, Se^4+^ induced cell differentiation and synergistically promoted As^3+^-induced cell differentiation. Mechanistic studies suggested that Se^4+^ might bind directly to PML-R in the form of divalent selenium (Se^2+^) to promote the degradation of PML–RAR*α* oncoprotein.

## Results

### Effects of Se^4+^ and As^3+^ on the growth of NB4 cells

**Figure 1:**
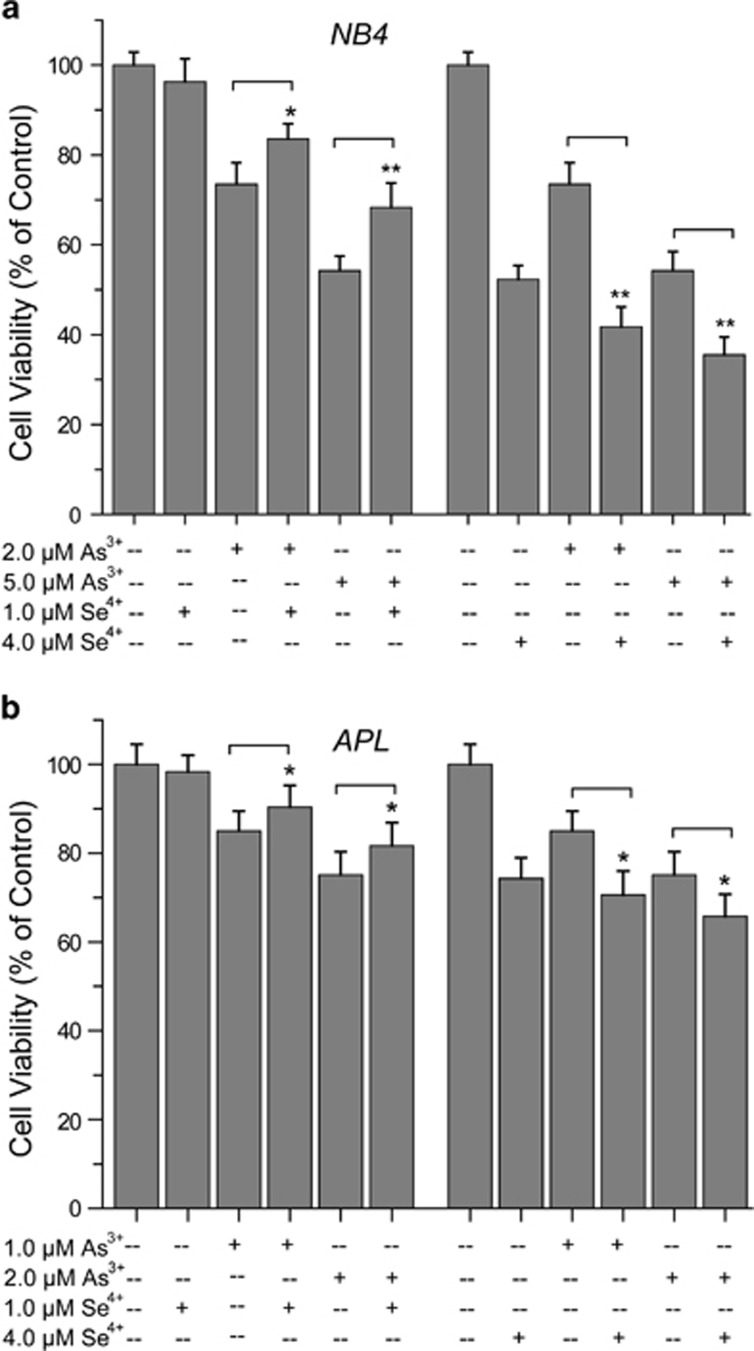
Effects of Se^4+^ on As^3+^-induced cell death. (**a**) The viability of NB4 cells was determined with the WST-1 cell proliferation assay after 48 h of treatment. (**b**) The viability of primary APL cells. Error bars represent S.D. from the mean of three separate experiments. *P<0.05 and ^**^P<0.01 compared with As^3+^-treated cells

After 48 h of treatment, cells viability was determined by the Trypan blue exclusion test.^20^ The viability of NB4 cells was 98%, and the viability of primary APL cells was 96%. The effects of As^3+^, Se^4+^, or their combination on the growth of NB4 cells and primary APL cells were determined by WST-1 cell proliferation assay (Figure 1). Se^4+^ exerted dose-dependent effects on NB4 cell proliferation. Se^4+^ at 4.0 *μ*M significantly inhibited the growth of NB4 cells, but 1.0 *μ*M Se^4+^ did not have this effect. In addition, 1.0 *μ*M Se^4+^ markedly reduced the inhibitory effects of As^3+^ on NB4 cell growth, whereas 4.0 *μ*M Se^4+^ enhanced the cell death induced by As^3+^ (Figure 1a). The viability of primary APL cells (%) in response to As^3+^, Se^4+^, or their combination was also investigated. Similarly, 4.0 *μ*M Se^4+^ inhibited the proliferation of primary APL cells and enhanced As^3+^-induced cell death (Figure 1b).

### Effects of Se^4+^ on As^3+^-induced cell apoptosis

**Figure 2:**
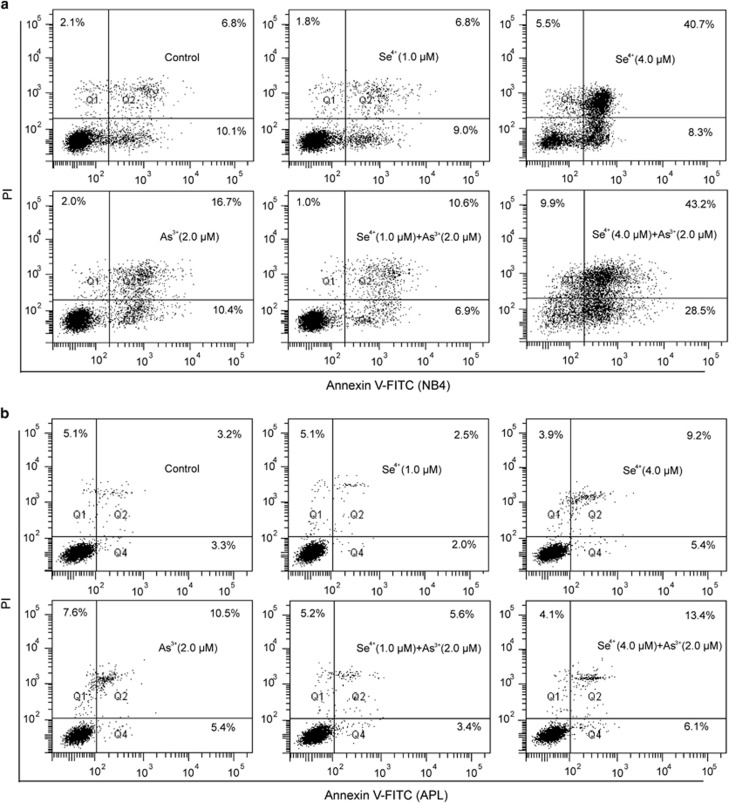
Effects of As^3+^ and Se^4+^ on cell apoptosis. (**a**) Cell apoptosis in NB4 cells measured by Annexin V-FITC and PI double staining. (**b**) Cell apoptosis in primary APL cells. Q_1_ and Q_3_ respectively represent the proportions of dead cells and living cells, and Q_2_ and Q_4_ were used to calculate apoptotic cells. Figures show a representative experiment from three independent experiments

Concentrations of 1.0 and 4.0 *μ*M Se^4+^ were used to investigate the effects of Se^4+^ on As^3+^-induced apoptosis in NB4 cells and primary APL cells. After 48 h of treatment, 2.0 *μ*M As^3+^ promoted the apoptosis of NB4 cells (Figure 2a). Compared with control, 1.0 *μ*M Se^4+^ decreased the percentage of apoptotic cells from 17.9 to 15.8%, but 4.0 *μ*M Se^4+^ increased the percentage from 17.9 to 49.0% (Figure 2a). Similar to the effects of Se^4+^ on As^3+^-induced cell death, 1.0 *μ*M Se^4+^ inhibited As^3+^-induced apoptosis in NB4 cells, but 4.0 *μ*M Se^4+^ enhanced apoptosis (Figure 2a). We also investigated the effects of 1.0 and 4.0 *μ*M Se^4+^ on the apoptosis of primary APL cells (Figure 2b). The potency of Se^4+^ and As^3+^ in inducing primary APL cell apoptosis was low, but the characteristics were similar. At 1.0 *μ*M, Se^4+^ inhibited As^3+^-induced apoptosis from 15.9 to 9.0%, whereas 4.0 *μ*M Se^4+^ enhanced As^3+^-induced apoptosis from 15.9 to 19.5% (Figure 2b).

### Effects of Se^4+^ and As^3+^ on cell cycle distribution

**Figure 3:**
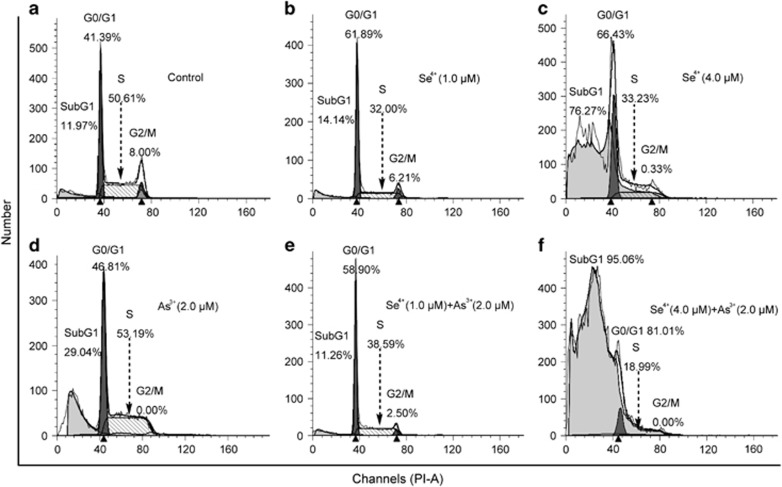
Cell cycle distribution profiles of NB4 cells. (**a**) Cell cycle distribution of NB4 cells analyzed by flow cytometry. (**b**) Se^4+^ at 1.0 *μ*M and (**c**) 4.0 *μ*M arrested the G_1_/S transition. (**d**) Effects of As^3+^ on the cell cycle. (**e**) Effects of 1.0 *μ*M Se^4+^ combined with 2.0 *μ*M As^3+^ on the cell cycle distribution. (**f**) Effects of 4.0 *μ*M Se^4+^ and 2.0 *μ*M As^3+^ on the cell cycle distribution. SubG_1_ represents the apoptotic cells. Figures show a representative experiment from three independent experiments

The cell cycle is a highly regulated event that controls the growth and differentiation of cells.^24, 25^ Changes in cell cycle distribution might be associated with the apoptosis and differentiation of NB4 cells. Thus, we analyzed the effects of As^3+^ and Se^4+^ on the cell cycle distribution (Figure 3). As^3+^ increased the level of SubG_1_ cells and blocked the G_1_/S transition (Figure 3d). Compared with control, the proportion of SubG_1_ cells in 4.0 *μ*M Se^4+^-treated cells increased from 11.97 to 76.27%, whereas the proportion in 1.0 *μ*M Se^4+^-treated cells was not obviously changed. However, both 1.0 and 4.0 *μ*M Se^4+^ significantly inhibited the G_1_/S transition and arrested the cell cycle at the G_0_/G_1_ phase (Figures 3a–c). Similarly, 1.0 *μ*M Se^4+^ decreased the proportion of SubG_1_ cells induced by As^3+^ from 29.04 to 11.26%, but 4.0 *μ*M Se^4+^ increased this proportion from 29.04 to 95.06% (Figures 3d–f). Compared with the As^3+^-treated group, low concentrations (1.0 and 4.0 *μ*M) of Se^4+^ enhanced the inhibition of As^3+^ in the G_1_/S transition (Figures 3d–f).

### Effects of Se^4+^ on arsenic uptake

**Figure 4:**
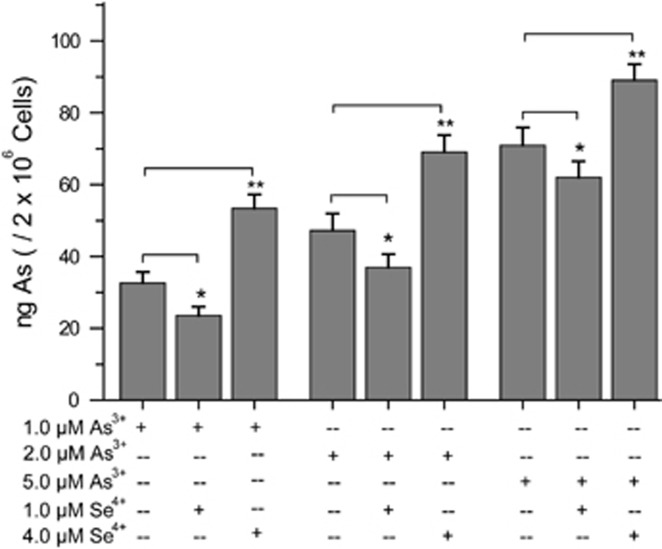
Effects of Se^4+^ on NB4 cell uptake of arsenic. Error bars represent S.D. from the mean of three independent experiments. *P<0.05 and ^**^P<0.01 compared with As^3+^-treated groups at corresponding concentrations

Inductively coupled plasma-mass spectrometry (ICP-MS) was used to detect arsenic in NB4 cells.^26, 27^ As shown in Figure 4, 1.0 and 4.0 *μ*M Se^4+^ inhibited and promoted the uptake of As^3+^ respectively, and this result can explain the dose-dependent contrasting effects of these concentrations of Se^4+^ on As^3+^-induced apoptosis.

### Effects of Se^4+^ and As^3+^ on cellular ROS

**Figure 5:**
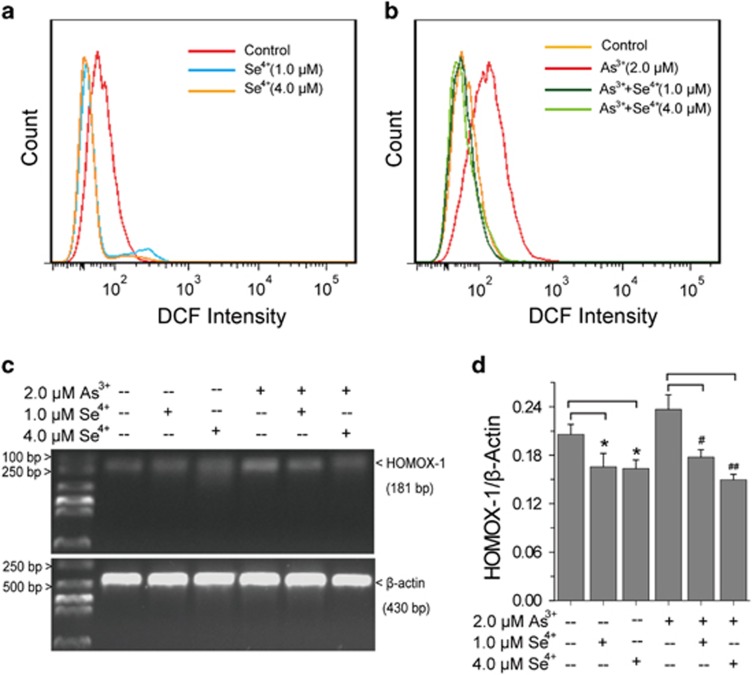
Effects of Se^4+^ on As^3+^-induced accumulation of ROS in NB4 cells. (**a**) Effects of Se^4+^ on ROS analyzed by flow cytometry. (**b**) Effects of Se^4+^ on As^3+^-induced accumulation of ROS in NB4 cells. (**c**) RT-PCR analysis of the expression of HMOX1 induced by Se^4+^, As^3+^, or their combination. (**d**) Percentage of relative intensity obtained from the corresponding RT-PCR. Error bars represent S.D. from the mean of three independent experiments. *P<0.05 compared with control, ^#^*P*<0.05 and ^##^*P*<0.01 compared with As^3+^-treated cells

Cellular ROS were detected using a fluorescence probe 2′,7′-dichlorodihydrofluorescein diacetate (DCFH-DA) by flow cytometry (Figure 5).^28^ Both 1.0 and 4.0 *μ*M Se^4+^ decreased the level of cellular ROS (Figure 5a). However, As^3+^ significantly increased cellular ROS after 36 h of treatment. Adding Se^4+^ eliminated the ROS generated by As^3+^ (Figure 5b). The expression of heme oxygenase-1 (HMOX1), a key oxidative stress response enzyme that is upregulated in the presence of elevated ROS, was analyzed by RT-PCR (Figure 5c).^29, 30^ As^3+^ upregulated the expression of HMOX1, but Se^4+^ alone had no significant effect. Furthermore, Se^4+^ inhibited the upregulation of HMOX1 induced by As^3+^ (Figure 5d).

### Effects of Se^4+^ and As^3+^ on the expression of apoptotic factors

**Figure 6:**
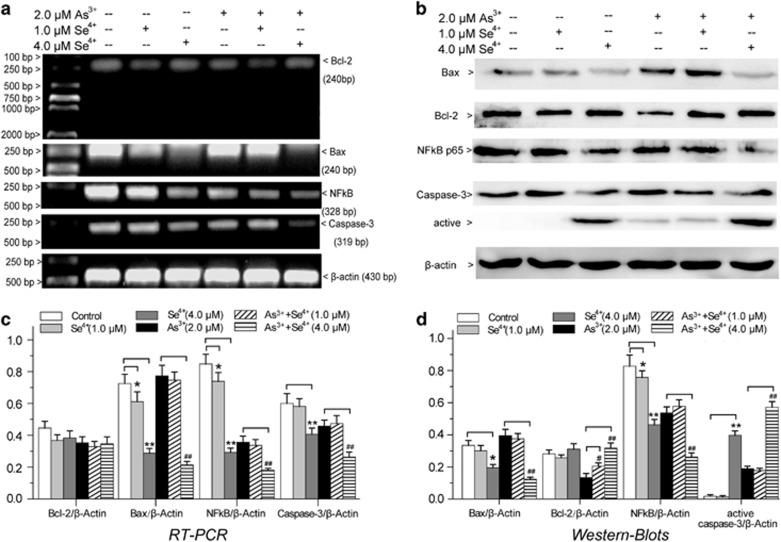
The effects of Se^4+^ and As^3+^ on apoptotic factors in NB4 cells. (**a**) Effects of As^3+^, Se^4+^, and their combination on the expression of Bax, Bcl-2, NF-*к*B, and Caspase-3 were analyzed by RT-PCR. (**b**) Effects of drugs on the expression of apoptosis factors were analyzed by western blots. (**c**) Relative intensity expression obtained from the corresponding RT-PCR and (**d**) western blots. Error bars represent S.D. from the mean of three separate experiments. *P<0.05 and ^**^P<0.01 compared with control, ^#^*P*<0.05 and ^##^*P*<0.01 compared with As^3+^-treated cells

We analyzed the expression of key apoptotic factors by RT-PCR and western blot (Figure 6). As^3+^ upregulated the Bax pro-apoptotic factor and downregulated the Bcl-2 anti-apoptotic factor at both gene and protein levels (Figures 6a and b). Se^4+^ (1.0 and 4.0 *μ*M) did not obviously regulate the expression of Bcl-2, but it downregulated the expression of Bax. Moreover, Se^4+^ (1.0 and 4.0 *μ*M) showed antagonistic effects with As^3+^ on the regulation of Bax and Bcl-2 (Figures 6c and d). The results suggested that Se^4+^ inhibited the mitochondria-mediated apoptosis.^13^

To clarify the mechanism of Se^4+^-induced apoptosis, we studied the effects of Se^4+^ on the expression of NF-*к*B and caspase-3 (Figure 6). Se^4+^ (1.0 and 4.0 *μ*M) obviously downregulated the expression of NF-*кB*. Se^4+^ at 4.0 *μ*M induced the activation of caspase-3, but 1.0 *μ*M Se^4+^ did not have this effect. Moreover, we found that 4.0 *μ*M Se^3+^ enhanced the regulation of As^3+^ in the expression of NF-*к*B and caspase-3 (Figure 6).

### Effects of Se^4+^ and As^3+^ on cell differentiation

**Figure 7:**
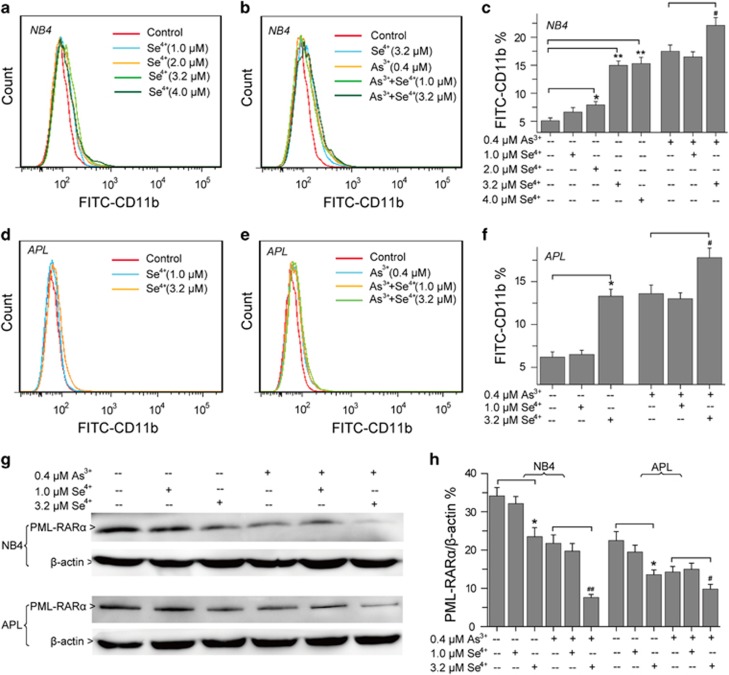
Cell differentiation and the fate of PML–RAR*α* oncoprotein. (**a**) Effects of Se^4+^ on the differentiation of NB4 cells were analyzed using FITC anti-human CD11b antibody with flow cytometry. (**b**) Effects of Se^4+^ and As^3+^ on the differentiation of NB4 cells. (**c**) Proportions of FITC-CD11b-positive NB4 cells. (**d**) Effects of Se^4+^ on the differentiation of primary APL cells. (**e**) Effects of combined Se^4+^ and As^3+^ on the differentiation of primary APL cells. (**f**) Proportions of FITC-CD11b-positive primary APL cells. (**g**) Expression of PML–RAR*α* fusion protein analyzed by western blot. (**h**) Relative intensity expression obtained from corresponding western blot. Error bars represent S.D. from the mean of three separate experiments. *P<0.05 and ^**^P<0.01 compared with control, ^#^*P*<0.05 and ^##^*P*<0.01 compared with As^3+^-treated cells

The differentiation of NB4 cells and primary APL cells were investigated by FITC anti-human CD11b antibody (Figure 7). At 2.0–4.0 *μ*M, Se^4+^ induced the differentiation of NB4 cells, and the numbers of FITC-CD11b-positive cells were obviously increased (Figures 7a and c). Compared with 0.4 *μ*M As^3+^ alone, 3.2 *μ*M Se^4+^ enhanced the As^3+^-induced differentiation of NB4 cells (Figures 7b and c). In consideration of the difference between NB4 cells and primary APL cells, we investigated the effects of Se^4+^ and As^3+^ on the expression of CD11b in primary APL cells (Figures 7d–f). Se^4+^ at 3.2 *μ*M obviously increased the number of FITC-CD11b-positive cells (Figures 7d–f). Meanwhile, Se^4+^ and As^3+^ synergistically promoted the differentiation of primary APL cells (Figures 7e and f). Considering that the differentiation of NB4 cells and primary APL cells is associated with the degradation of PML–RAR*α* fusion protein, we analyzed the expression of this oncoprotein by western Blot. After 96 h of treatment, 3.2 *μ*M Se^4+^ dramatically induced the degradation of PML–RAR*α* oncoprotein (Figures 7g and h). Se^4+^ at 1.0 *μ*M inhibited As^3+^-induced degradation of PML–RAR*α* fusion protein, whereas 3.2 *μ*M Se^4+^ acted synergistically with As^3+^ (0.4 *μ*M) to promote the degradation of PML–RAR*α* oncoprotein (Figures 7g and h).

### Interactions between Se^4+^ and PML-R

**Figure 8:**
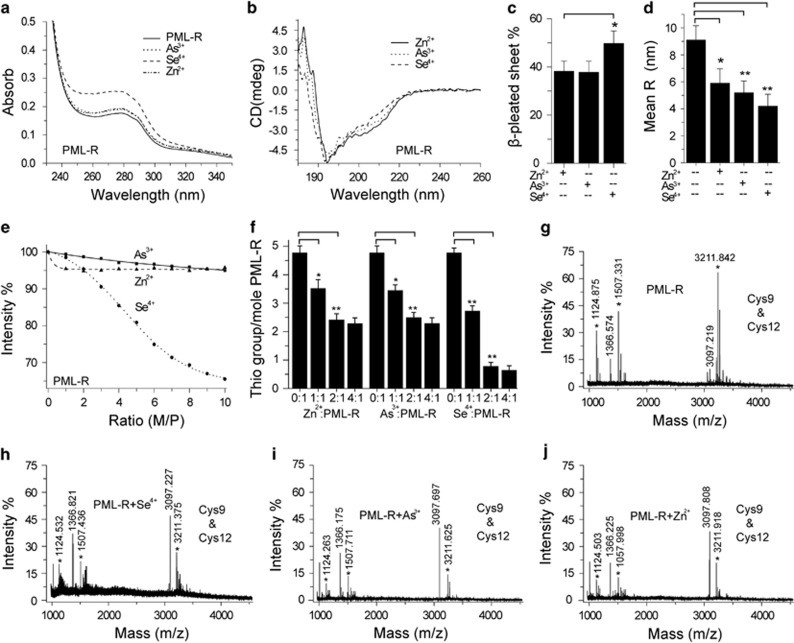
Interactions between Se^4+^ and PML-R. (**a**) UV spectra for the effects of Zn^2+^, As^3+^, and Se^4+^ on the absorbance of PML-R at 280 nm. (**b**) CD spectra for the effects of Zn^2+^, As^3+^, and Se^4+^ on the conformation of PML-R. (**c**) Changes of *β*-pleated sheet calculated according to CD spectra, ^**^P<0.01 compared with the other two. (**d**) Effects of Zn^2+^, As^3+^, and Se^4+^ on the R_H_ of PML-R measured by DLS, ^**^P<0.01 compared with Zn^2+^-treated group. (**e**) Synchronous fluorescence spectra (Δ*λ*=60 nm) for the interactions between metal/semimetal ion and PML-R at 285 nm. (**f**) Thiol groups of PML-R determined by Ellman's test, *P<0.05 and ^**^P<0.01 compared with control. (**g**) Peaks corresponding to Cys9 and Cys12 were detected in PML-R by MALDI-TOF-MS. (**h**) Effects of Se^4+^, (**i**) As^3+^, and (**j**) Zn^2+^on Cys residues 9 and 12. *Represents IA-modified fragments. The molar ratio of metal ion to protein is 2 : 1. Figures show a representative experiment from three independent experiments

To investigate whether Se^4+^ promotes the catabolism of PML–RAR*α* oncoprotein by directly interacting with PML-R, we analyzed the interactions between Se^4+^ and PML-R. The intrinsic ultraviolet–visible (UV–vis) absorption peak of PML-R at 280 nm is primarily caused by Trp47, and the intensity of this peak can indicate perturbation of the microenvironment around Trp47.^17, 31^ After incubation with Se^4+^ for 15 min, the intensity of the 280 nm peak was increased. Compared with Zn^2+^ and As^3+^, Se^4+^ increased the intensity at 280 nm more obviously (Figure 8a). The conformational changes of PML-R were also detected by circular dichroism (CD).^27^ The conformation of the PML-R zinc-finger domain was disordered.^17^ Zn^2+^ induced PML-R folding to a stable structure (Figure 8b). Similarly, Se^4+^ and As^3+^ promoted the folding of PML-R (Figure 8b). Compared with Zn^2+^ and As^3+^, Se^4+^ evidently increased the *β*-pleated sheet of PML-R (Figures 8b and c). As evidenced by the hydrodynamic radius (R_H_) of PML-R analyzed by dynamic light scattering (DLS), PML-R was in an unfolded state. Se^4+^ induced the folding of PML-R,^32^ because R_H_ was decreased from 9.1 to 4.3 nm (Figure 8d). Changes in the maximum emission wavelength and intensity of the synchronous fluorescence spectrum (Δ*λ*=60 nm) can reflect the microenvironment around Trp47 for PML-R.^17, 33^ Se^4+^ dramatically decreased the synchronous fluorescence intensity of PML-R at 285 nm with an increasing mole ratio, but Zn^2+^ and As^3+^ slightly decreased this fluorescence (Figure 8e). Spectrographic analysis suggested that Se^4+^ and As^3+^ promoted the folding of the PML-R zinc-finger domain. Differently, Se^4+^ affected the conformation of PML-R more remarkably.

Zhang *et al.*^16^ reported that Cys residues in PML-R-ZFs were involved in the binding of As^3+^. To determine the mechanism of Se^4+^ binding, the effects of Se^4+^ on thiol groups of PML-R were analyzed by the method of Ellman.^27, 31^ A total of 4.76 (thiol group/mol of protein) thiol groups were detected in PML-R (Figure 8f). Unlike Zn^2+^ and As^3+^, Se^4+^ dramatically eliminated the thiol groups in PML-R, indicating stronger coordination with Cys residues (Figure 8f). Subsequently, the Cys residues involved in selenium binding were detected by matrix-assisted laser desorption ionization-time-of-flight (MALDI-TOF)-MS. After being incubated with Zn^2+^, As^3+^, or Se^4+^ and then alkylated with iodoacetamide (IA), PML-R was digested by trypsin for analysis. The peaks corresponding to Cys9 (IA-modified) and Cys12 (IA-modified) were detected (Figure 8g). Compared with PML-R, adding Zn^2+^, As^3+^, or Se^4+^ decreased the intensities of these two peaks (Figures 8g–j).

## Discussion

The chemical properties and metabolic fates of selenium are similar to those of arsenic.^23^ Uniquely, selenium is an essential nutrient element that shows lower genotoxicity, cytotoxicity, and oxidative toxicity.^18^ As ATO has been successfully used in the treatment of APL, combination therapy has been advocated.^34^ Arsenic generates ROS and induces oxidative damage that limits its application in the treatment of diseases, including cancers.^6, 35^ However, low concentrations of selenium can eliminate ROS and protect organisms.^18^ In consideration of the complicated interaction between selenium and arsenic,^23^ the co-effects of low doses of Se^4+^ and As^3+^ on the apoptosis and differentiation of APL cells were evaluated.

Bcl-2, Bax, NF-кB, and caspase-3, which play key roles in ATO-induced cell apoptosis,^10, 14, 15^ were selected to study the mechanism for the apoptosis of NB4 cells induced by As^3+^ (2.0 *μ*M), Se^4+^ (1.0 and 4.0 *μ*M), or a combination of As^3+^ and Se^4+^. As^3+^ arrested the G_1_/S transition, increased the cellular ROS, upregulated the Bax pro-apoptotic factor, downregulated the Bcl-2 and NF-*к*B anti-apoptotic factors, and activated caspase-3. These changes of NB4 cells suggested that As^3+^ induced the apoptosis of NB4 cells by promoting the mitochondria-mediated intrinsic pathway and inhibiting the NF-*к*B pathway.^10, 13, 14, 15^

Concentrations of Se^4+^ greater than 2.0 *μ*M inhibit the growth of NB4 cells.^19^ However, when the concentration of selenium increased to a certain extent, it will induce the accumulation of ROS *in vivo* and lead to adverse effects.^22^ In this work, 1.0–4.0 *μ*M Se^4+^ inhibited the generation of cellular ROS and blocked mitochondria-mediated apoptosis by downregulating the expression of Bax and upregulating the expression of Bcl-2. However, Se^4+^ arrested the cell cycle at G_0_/G_1_, downregulated the expression of NF-*к*B, and induced the activation of caspase-3 to promote NB4 cell apoptosis. Because the promoting effects exceeded the inhibitory effects, 4.0 *μ*M Se^4+^ induced the apoptosis of NB4 cells.

At 1.0 *μ*M, Se^4+^ inhibited As^3+^ uptake, eliminated As^3+^-generated ROS, and prevented the cells from undergoing As^3+^-induced apoptosis by increasing the expression of Bcl-2. Contrarily, 4.0 *μ*M Se^4+^ enhanced As^3+^-induced apoptosis and arrested the cell cycle at the G_0_/G_1_ phase. On one hand, 4.0 *μ*M Se^4+^ promoted the uptake of As^3+^ and enhanced the downregulation of NF-*к*B and the activation of caspase-3 that were induced by As^3+^. On the other hand, 4.0 *μ*M Se^4+^ inhibited the apoptosis of NB4 cells by eliminating As^3+^-generated ROS and inhibiting the regulatory effects of As^3+^ toward the Bax pro-apoptotic factor and the Bcl-2 anti-apoptotic factor. In general, As^3+^-induced apoptosis was more prone to promotion by 4.0 *μ*M Se^4+^ than to inhibition.

In addition to apoptosis, 2.0–4.0 *μ*M Se^4+^ induced the differentiation of NB4 cells and primary APL cells. The combination of 3.2 *μ*M Se^4+^ and 0.4 *μ*M As^3+^ enhanced the differentiation of NB4 cells. The PML–RAR*α* fusion protein is the key driver of APL leukemogenesis and the target of ATO.^2^ The differentiation of human APL cells induced by ATO is related to the degradation of PML–RAR*α* fusion protein.^16^ In consideration of the similarity between arsenic and selenium, we hypothesized that Se^4+^-induced differentiation of NB4 cells and primary APL cells might be related to the degradation of PML–RAR*α* fusion protein. The results of western blot confirmed the hypothesis that Se^4+^ caused the decomposition of PML–RAR*α* oncoprotein in both NB4 cells and primary APL cells.

The Cys-rich zinc-finger domain of PML-R is the binding domain of As^3+^.^16^ Similar to As^3+^, Se^4+^ was readily bound to thiol groups *in vitro*.^36^ To investigate whether Se^4+^ directly binds to PML-R, the interactions between Se^4+^ and PML-R were studied. The zinc-finger domain of PML-R contains two PML-R-ZFs.^17^ The spectroscopic data herein showed that Zn^2+^, As^3+^, and Se^4+^ induced the folding of PML-R and exposed the residues of Cys24, His26, Cys40, and Cys43 near Trp47 in PML-R-ZF2. Compared with Zn^2+^ and As^3+^, Se^4+^ induced conformational changes of PML-R evidently and uniquely. In addition, more thiol groups in PML-R were involved in the binding of Se^4+^. GSSeSG is another selenium substrate for proteins that formed by the reduction of Se^4+^ with glutathione.^36, 37^ Shi *et al.*^35^ reported that Se^2+^ was the terminal form that bound to Cys-rich proteins. In the binding reaction, one Se^4+^ needed four Cys residues. Two Cys residues were oxidized to a Cys–Cys pair in the process of reducing Se^4+^, whereas the other two bound to the reduced Se^2+^ in the form of RSSeSR.^35^ The *in vitro* experiments on the interaction between Se^4+^ and PML-R suggested that Se^4+^ might be reduced to Se^2+^ that then bound PML-R. The large conformational changes of PML-R might be ascribed to the formation of disulfide bonds. Moreover, MALDI-TOF-MS spectra showed that Cys9 and Cys12 at PLM-R-ZR1 were involved in the binding of Se^4+^. Therefore, Se^2+^ might be the form of selenium that promoted the *in vivo* degradation of PML–RAR*α* fusion protein by directly binding to PML-R-ZFs.

**Figure 9:**
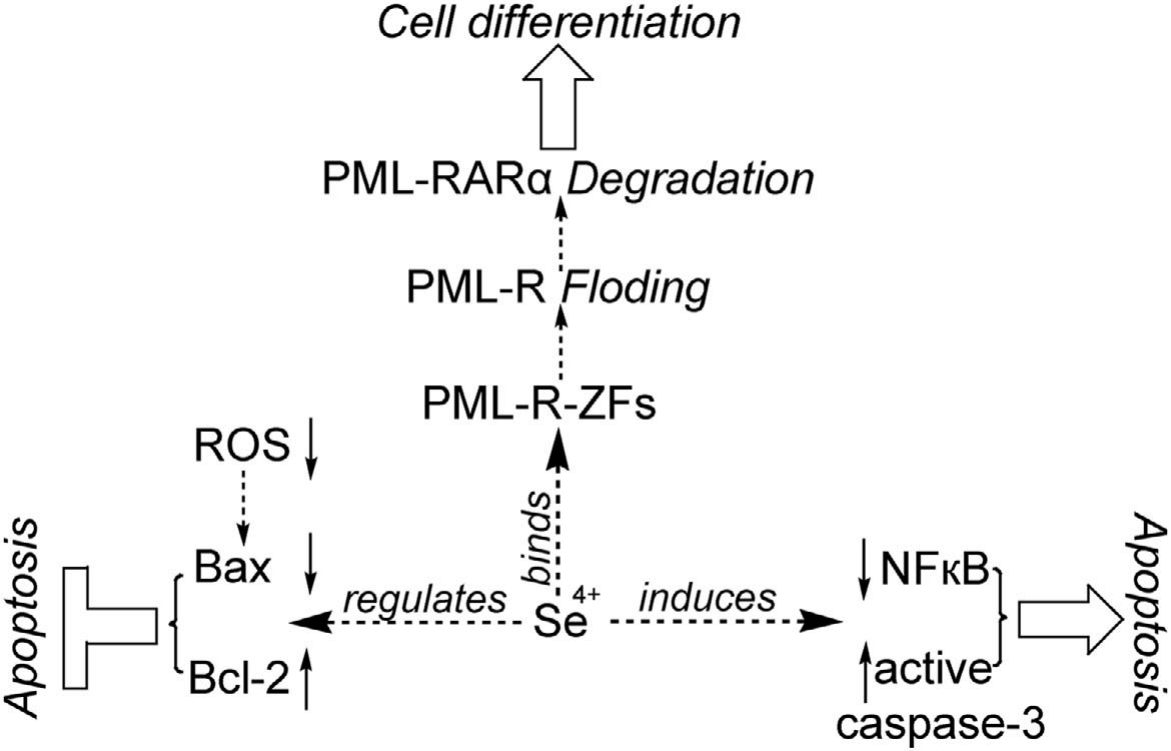
Mechanism for the effects of Se^4+^ (1.0 and 4.0 *μ*M) on As^3+^-induced apoptosis and differentiation in NB4 cells and primary APL cells. Se^4+^ promotes the degradation of PML–RAR*α* fusion protein by directly binding to the PML-R-ZFs. The decomposition of PML–RAR*α* oncoprotein contributes to the differentiation of NB4 cells and primary APL cells. On one hand, Se^4+^ (4.0 *μ*M) prevents the cells from undergoing As^3+^-induced apoptosis by eliminating ROS, downregulating the Bax pro-apoptotic factor, and upregulating the Bcl-2 anti-apoptotic factor. On the other hand, Se^4+^ enhances As^3+^-induced apoptosis by downregulating NF-*ĸ*B and activating caspase-3

In summary, the mechanism for the effects of Se^4+^ on As^3+^-induced apoptosis and differentiation in NB4 cells and primary APL cells was postulated. As shown in Figure 9, Se^4+^ at low concentrations (1.0 and 4.0 *μ*M) showed contrasting effects on As^3+^-induced apoptosis in NB4 cells and primary APL cells. On one hand, Se^4+^ (1.0 and 4.0 *μ*M) inhibited the mitochondria-mediated intrinsic apoptosis by eliminating As^3+^-generated ROS. On the other hand, Se^4+^ (4.0 *μ*M) promoted As^3+^-induced apoptosis by facilitating the downregulation of NF-*ĸ*B and the activation of caspase-3. The effects of Se^4+^ on As^3+^-induced differentiation in NB4 cells and primary APL cells were caused by the degradation of PML–RAR*α* oncoprotein. Thus, Se^4+^, which is similar to As^3+^, might directly bind to PML-R in the form of Se^2+^ to control the fate of PML–RAR*α* fusion protein. In the meantime, Cys9 and Cys12 in PML-R-ZF1 are involved in the binding reaction.

## Materials and Methods

### Caution

Safeguards are required to mitigate the potential risk of arsenic compounds.^35^

### Chemicals and antibodies

BSA, NaAsO_2_, Na_2_SeO_3_, and anti-PML rabbit mAb were purchased from Sigma-Aldrich (St. Louis, MO, USA). Anti-Bcl-2 (50E3) rabbit mAb, anti-Bax (D2E11) rabbit mAb, anti-NF-*к*B p65 (D14E12) XP rabbit mAb, and anti-Caspase-3 (8G10) rabbit mAb were purchased from Cell Signaling Technology (Boston, MA, USA). FITC anti-human CD11b antibody was obtained from BioLegend (San Diego, CA, USA). Anti-*β*-Actin mouse mAb was purchased from Beyotime (Nantong, China).

### Cell culture and cell viability assay

Human NB4 leukemia cells used in the experiments were purchased from SXBIO Biotech, Shanghai, China). NB4 cells were cultured in RPMI-1640 (KeyGEN Biotech, Nanjing, China) with 10% fetal bovine serum (FBS) at 37 °C under a 5% CO_2_ atmosphere. After informed consent, the bone marrow of two primary APL patients was acquired from DrumTower Hospital (Nanjing, China). Human primary APL cells were separated from the bone marrow by traditional Ficoll-Hypaque density centrifugation.^38^ The fresh primary APL cells were cultured in RPMI-1640 (KeyGEN Biotech) with 15% FBS at 37 °C under a 5% CO_2_ atmosphere. The viability of NB4 cells and primary APL cells were determined by the Trypan blue exclusion method.^20^ After culturing for 24, 48, and 96 h, cells were collected and mixed with equal volume of PBS containing 0.4% Trypan blue dye. Cell viability was calculated as the number of viable cells divided by the total number of cells with the grids on the hemacytometer. The effects of Se^4+^ and As^3+^ on cell growth were measured with the WST-1 cell proliferation assay kit according to the manufacturer's protocols (KeyGEN Biotech).^39^ The cells were seeded at 4 × 10^4^ cells/ml in a 96-well culture plate and then exposed to various concentrations of As^3+^, Se^4+^, or their combination for 48 h. Untreated cells served as controls.

### Preparation of PML-R

The gene for PML-R was synthesized from Invitrogen Life Technologies (Carlsbad, CA, USA) and cloned into the *Nco*I–*Bam*HI restriction sites of the pET-28a vector (Novagen, San Diego, CA, USA). The expression plasmid pET-28a-*PML-R* was confirmed by DNA sequencing. *E. coli* BL21 (DE3) pLysS was transformed by the ligated plasmid by heat shock at 42 °C for 30 s. After being selected on standard kanamycin-containing agar plates, the colonies were expressed at 22 °C for 10 h. The separation and purification of PML-R was similar to our previous work.^40^ The concentration of PML-R was determined by a Bradford assay based on a BSA standard curve.^31^

### Analysis of apoptotic cells by flow cytometry

NB4 cells and primary APL cells were treated with As^3+^ (2.0 *μ*M), Se^4+^ (1.0 or 4.0 *μ*M), or their combination for 48 h. After treatment, the cells were collected, washed twice with Ca^2+^-free PBS, and stained with Annexin V-FITC and propidium iodide (PI). After double staining, the cells were recorded on a BD LSRL Fortessa flow cytometer (Franklin Lakes, NJ, USA).^39^ The percentages of apoptotic cells were calculated by BD FACSDiva software (Franklin Lakes, NJ, USA).

### Analysis of cellular ROS and cell cycle by flow cytometry

NB4 cells were treated with As^3+^ (2.0 *μ*M), Se^4+^ (1.0 or 4.0 *μ*M), or their combination for 36 h. Cellular ROS were assessed with the DCFH-DA fluorescent probe.^28^ The levels of cellular 2′,7′-dichlorofluorescein (DCF) were positively correlated with ROS. After treatment, NB4 cells were washed twice with PBS and incubated in RPMI-1640 medium containing 10 *μ*M DCFH-DA at 37 °C for 30 min. The cells were then washed twice to remove excess probes for further analysis. The percentages of DCF-positive cells were analyzed by FlowJo 7.6 (Franklin Lakes, NJ, USA). Cell cycle distribution was determined by PI staining. After 36 h of treatment, NB4 cells were washed twice with Ca^2+^-free PBS and then fixed with 70% ethanol at 4 °C for 18 h. To extract low-molecular-weight DNA from cell nuclei, the fixed NB4 cells were digested in 0.5 mg/ml RNase (Sigma, St. Louis, MO, USA) containing PBS at 37 °C for 30 min. The remnant DNA in cells was stained with 0.05 mg/ml PI and reacted in dark at room temperature for 30 min. The data of cell cycle distribution were analyzed by ModFit LT 3.3 software (Franklin Lakes, NJ, USA). Apoptotic and nonapoptotic cells were counted on the basis of DNA content.^39^

### Analysis of FITC-CD11b-positive cells by flow cytometry

The differentiation of NB4 cells and primary APL cells was determined with a FITC anti-human CD11b antibody.^10^ After 96 h of treatment, cells were washed twice with PBS and counted. A total of 1 × 10^6^ cells in 100 *μ*l of PBS was incubated with 20 *μ*l FITC anti-human CD11b antibody at 4 °C for 30 min. Excess antibody was washed out by PBS. These data were analyzed by BD FACSDiva software.

### Measurement of cellular arsenic concentration

After co-treatments with Se^4+^ (1.0 or 4.0 *μ*M) and As^3+^ (1.0, 2.0, or 5.0 *μ*M) for 48 h, NB4 cells were washed twice with PBS, counted, and harvested in 1.0 ml of mixture containing 0.4 ml H_2_O_2_ and 0.6 ml Tris-HNO_3_ (50 mM, pH 7.4) for digestion. Digested samples were filtered through a 0.22 *μ*m pore membrane and diluted with deionized water for analysis. The concentrations of arsenic were determined by ICP-MS (ELAN 9000, Waltham, MA, USA).^26^

### RT-PCR analysis

Total RNA was extracted from NB4 cells by RNAiso Plus (Takara, Dalian, China). Isolated total RNA (2 *μ*g) was used to perform the reverse transcription with the PrimeScript RT reagent kit (Takara) according to the manufacturer's protocols.^39^ The transcribed cDNA (2 *μ*l) was used for PCR amplification with specific primers for HMOX1, caspase-3, Bax, Bcl-2, NF-*к*B, and *β*-Actin genes. Thirty cycles were performed under the following conditions: 30 s denaturation at 94 °C, 30 s annealing at 52 °C (*β*-Actin), 57 °C (HMOX1, Bax, Bcl-2, NF-*к*B), or 51 °C (caspase-3), and 30 s extension at 72 °C. The PCR products were separated on 1% agarose gels containing ethidium bromide. The separated bands were photographed on a Gel Doc XR System (Bio-Rad, Hercules, CA, USA). Primer sequences are shown in Table 1.

### Western blot analysis

Total protein in NB4 cells and primary APL cells was extracted by ice-cold RIPA cell lysis buffer (Beyotime). The concentration of proteins was determined by a BCA protein quantification kit (Beyotime). Total protein (30 *μ*g) from each sample was separated by 12% sodium dodecyl sulfate-polyacrylamide gel electrophoresis and then transferred onto a PVDF membrane (Millipore, Billerica, MA, USA). After blocking in 5% skim milk at room temperature for 1 h, the membrane was sequentially incubated with primary and secondary antibodies. Proteins on the PVDF membrane were visualized using chemiluminescent HRP substrate (Millipore).^39^ The intensities of the bands were normalized to that of *β*-Actin. All the experiments were repeated three or more times.

### Spectrographic analysis of the interactions between Se^4+^/As^3+^/Zn^2+^ and PML-R

Zn^2+^, As^3+^, and Se^4+^ were each incubated with PML-R at room temperature for 15 min before spectrographic analysis. UV–vis spectra of PML-R were recorded on a Perkin Elmer Lambda-35 spectrophotometer (Waltham, MA, USA).^31^ CD spectra were measured by a JASCO-J810 spectropolarimeter (Jasco Co., Tokyo, Japan).^27^ The molar ratio of metal ion to PML-R was 2 : 1. Synchronous fluorescence spectra (Δ*λ*=60 nm) were recorded on a 48 000 DSCF time-resolved fluorescence spectrometer (SLM Co., Sunnyvale, CA, USA) equipped with 1.0 cm quartz cell.^27, 33^ The molar ratios of ion to PML-R were increased from 0 : 1 to 10 : 1. All spectra were obtained at room temperature.

### Dynamic light scattering analysis

The R_H_ of PML-R was determined by BI-200SM DLS (Brookhaven, Holtsville, NY, USA) at 25.0±0.1 °C. In the experiments, 0.2 mg/ml PML-R was incubated with ZnSO_4_, As^3+^, and Se^4+^ in H_2_O at 25 °C for 2 h, and then recorded on the instrument. The molar ratio of ion to protein was 2 : 1. Two replicates were tested for each sample. Data were automatically calculated by the dynamic light scattering software.^32^

### Thiol groups in PML-R analyzed by Ellman's test

The effects of Zn^2+^, As^3+^, and Se^4+^ on free thiol groups in PML-R were determined by the method of Ellman.^27, 31^ The reaction mixtures (200 *μ*l) containing 20 *μ*M PML-R and ion (Zn^2+^, As^3+^, or Se^4+^) were incubated at 37 °C for 2 h. The molar ratios of ion to PML-R were 0 : 1, 1 : 1, 2 : 1, and 4 : 1. After reaction, PML-R was incubated with 0.1 *μ*M 5,5′-dithiobis-(2-nitrobenzoic acid) (DTNB) in Tris-HCl buffer (20 mM, pH 8.0) at 25 °C for 30 min. The absorbance at 412 nm was detected on Perkin Elmer Lambda-35 spectrophotometer. Solutions containing ions (Zn^2+^, As^3+^, or Se^4+^) and 0.1 *μ*M DTNB were employed as controls. The concentration of free thiol groups was calculated with the standard curves of Cys.^31^ Two independent experiments were performed. The values represent a mean of three replicates.

### Cysteine residues in PML-R analyzed by MALDI-TOF-MS

Before analysis, the mixtures (0.5 ml) containing 20 *μ*M purified PML-R and 200 *μ*M ion (Zn^2+^, As^3+^, or Se^4+^) were incubated at 37 °C for 2 h. Then, Zn^2+^-, As^3+^-, or Se^4+^-containing solutions of PML-R were mixed with 50 mM IA in the dark at room temperature for 1 h. The residual IA and ions were washed out by NH_4_HCO_3_ (50 mM) using a centrifugal filter (3 kDa). The concentration of deionized PML-R was determined by the method of Bradford. The deionized PML-R was incubated with 5% trypsin overnight at 37 °C. Digested peptide fragments were dissolved in 60% acetonitrile and 0.1% (v/v) trifluoroacetic acid, lyophilized, and stored at −80 °C for analysis.^27, 41^

Before MS analysis, the lyophilized sample was dissolved in 10 *μ*l H_2_O and mixed with 10 *μ*l *α*-cyano-4-hydroxycinnamic acid matrix solution. The mixture was dried by spotting on a polished steel target plate. The masses of peptide fragments ware recorded on an AUTOFLEX II MALDI-TOF mass spectrometer (Bruker, Madison, WI,USA) with a 20-kV acceleration voltage.^40, 41^

### Statistical analysis

Two-tailed Student's *t*-tests were performed for comparisons of two groups. A *P-*value of <0.05 was considered statistically significant.

## Figures and Tables

**Table 1:** RT-PCR primer sequences

**Name**	**Sense**	**Antisense**
HMOX1	5′-CTTTGAGGAGTTGCAGGAGC-3′	5′-TGTAAGGACCCATCGGAGAA-3′
Bax	5′-TGACGGCAACTTCAACTGGG-3′	5′-AGCACTCCCGCCACAAAGA-3′
Bcl	5′-GGGAGGATTGTGGCCTTCTT-3′	5′-GGCCAAACTGAGCAGAGTCTTC-3′
NF-*к*B	5′-ACTACGAGGGACCAGCCAAGA-3′	5′-CGCAGCCGCACTATACTCA-3′
Caspase-3	5′-GTGGAATTGATGCGTGATG-3′	5′-AACCAGGTGCTGTGGAGTA-3′
*β*-Actin	5′-GACCTGACTGACTACCTC-3′	5′-TCTTCATTGTGCTGGGTGC-3′

## Abbreviations

As^3+^: arsenite
Se^4+^: selenite
ATO: arsenic trioxide
Se^2+^: divalent selenium
FBS: fetal bovine serum
NF*-к*B: nuclear factor-*к*B
ROS: reactive oxygen species
PML: promyelocytic leukemia protein
PML-R: PML RING domain
ZFs: Zn^2+^ binding sites
RAR*α*: retinoic acid receptor-*α*
APL: acute promyelocytic leukemia
DCFH-DA: 2′,7′-dichlorodihydrofluorescein diacetate
DTNB: 5,5′-dithiobis-(2-nitrobenzoic acid)
DCF: 2′,7′-dichlorofluorescein
IA: iodoacetamide
PI: propidium iodide
HMOX1: heme oxygenase-1
UV–vis: ultraviolet–visible
CD: circular dichroism
R_H_: hydrodynamic radius
DLS: dynamic light scattering
MALDI-TOF: matrix-assisted laser desorption ionization-time-of-flight
ICP: inductively coupled plasma
MS: mass spectrometry
